# Physiological Arousal and Emotion Regulation Strategies in Young Children with Autism Spectrum Disorders

**DOI:** 10.1007/s10803-017-3181-6

**Published:** 2017-06-07

**Authors:** Gemma Zantinge, Sophie van Rijn, Lex Stockmann, Hanna Swaab

**Affiliations:** 1 Department of Clinical Child and Adolescent Studies, Leiden University, Leiden, The Netherlands; 2 Leiden Institute for Brain and Cognition, Leiden, The Netherlands; 3 Centre for Autism, Rivierduinen, Leiden, The Netherlands

**Keywords:** Autism spectrum disorders, Emotion, Arousal, Cognition, Emotion regulation, Self-control

## Abstract

This study aimed to assess physiological arousal and behavioral regulation of emotion in the context of frustration in 29 children with Autism Spectrum Disorders (ASD) and 45 typically developing children (41–81 months). Heart rate was continuously measured and emotion strategies were coded, during a locked-box task. Results revealed increases in arousal followed by a decline during recovery, significant for both groups indicating that heart rate patterns between groups were identical. The ASD group deployed less constructive and more venting and avoidance strategies, which was related to language impairments. We conclude that rather than abnormal levels of emotional arousal, a key impairment in young children with ASD may be difficulties in behaviorally regulating and expressing experienced emotions to others.

## Introduction

Problematic emotional behavior as expressed in tantrums, irritability, aggression, self-injury, anxiety, and impulsivity is often reported by parents of children with Autism Spectrum Disorders (ASD) and professionals (Geller 2005; Lecavalier et al. 2006). These emotional behavior problems could be the consequence of compromised experience or expression of emotion. Abnormal levels of emotional arousal, deficient emotional control or a failure to adequately cope with emotions might help explain such emotional behavior problems.

Broadly defined, an emotion can be considered a complex psychological state involving a physiological response, a subjective feeling, and a behavioral response (Hockenbury and Hockenbury 2010). In typical development, adaptively coping with emotions can be helpful for decision making, motivation, and communication (Izard 1971, 2013) but emotions can also be hindering when the timing is off, when they are directed towards irrelevant aspects, or when the emotional intensity levels are not adapted to the situation (Lazarus 1991; Parrott 2001).

Problematic emotional behavior however, is not a diagnostic criterion for ASD, it is not exclusive to ASD, and over the past decades there has been little attention on emotion processing problems related to ASD in comparison to other domains such as social cognition. This has changed in recent years with an increased recognition that studying emotions may contribute substantially to the understanding of observed emotional and behavioral problems in ASD (Mazefsky et al. 2013). The majority of studies however, have focused on the ability of individuals with ASD to recognize emotions in others, rather than the experience and management of own emotions (Mazefsky et al. 2012). Also, studies have largely relied on behavioral observations and survey reports (Mazefsky et al. 2012) of emotional behavior. In addition to such behavioral measures of emotions, it is important to also include more sensitive and objective measures of the experienced emotions, such as physiological emotional arousal responses. The present study was designed to provide this.

Emotional arousal originates from the Autonomic Nervous System (ANS). The ANS consists of the parasympathetic nervous system (PNS; promoting calm, vegetative activities) and the sympathetic nervous system (SNS; involved in stress and activity), and is believed to be involved in affective, cognitive and behavioral responses of individuals (Benevides and Lane 2015). Heart rate is a commonly used measure of autonomic arousal, it varies due to influence and interaction between both the sympathetic (preparing the body for action) and parasympathetic activity (rest and digest) of the ANS (Benevides and Lane 2015). Arousal is crucial for steering and tuning our emotions and behavior in social situations in order to adapt and meet social goals (Chambers et al. 2009).

Research on arousal in children with ASD yielded mixed results (Benevides and Lane 2015). There seems to be evidence for normative baseline arousal, but deviating arousal levels in response to a variety of tasks and emotions of others (Benevides and Lane 2015; Rogers and Ozonoff 2005). However, caution should be taken because of the great variety in child characteristics and task design (Benevides and Lane 2015; Kreibig 2010). In addition, there is much less known about how young children with ASD experience emotions.

One way to study experienced emotions in young children is to elicit frustration (Kreibig 2010), which is a negative affect related to interruption of ongoing tasks or goal blocking (Rothbart 2007). Especially at this young age, blocking personal goals will inevitably trigger emotional arousal that is not only evident in a physiological response but also expressed in emotional behavior. Previous behavioral studies on emotion regulation in young children with ASD have shown that children with ASD deploy different coping strategies compared to typically developing children in the context of frustration (Jahromi et al. 2012; Samson et al. 2015). Children with ASD used significantly less constructive strategies (such as goal-directed behaviors, social support orienting, and verbal assistance seeking) but more venting (i.e. vocal and physical venting and self-speech) and avoidance (i.e. avoidance, distraction, and alternative behavior) strategies. However, there were no observed differences in facial or bodily negativity. The question is whether these expressed emotional behaviors originate from an inadequate arousal response. In other words, it is important to study both arousal and emotion regulation strategies, as these may be differentially affected in young children with ASD.

This study aims to integrate subjective, objective, and sensitive measures to investigate arousal and regulation of emotion in a sample of very young children with ASD in order to identify and understand possible differential mechanisms. Including physiological arousal measures (i.e. heart rate) in addition to behavioral measures could benefit the current need for knowledge about underlying mechanisms of daily life socio-emotional problems, which could be targeted in early intervention, and accompanying the advances in early detection of ASD (Bradshaw et al. 2015). The relevance of early intervention is further stressed by several studies showing that a vulnerability in emotional behavior has been associated with negative outcomes with respect to social functioning, depression, and anxiety later in life (Mazefsky 2015; Mazefsky et al. 2014; Nader-Grosbois and Mazzone 2014; Swain et al. 2015).

We hypothesize that children with ASD have more difficulties deploying adequate emotion coping strategies and show more venting and/or avoidance strategies. Given the previous literature on physiological arousal in children with ASD we expect a different arousal pattern in response to frustration. We also focus on characteristics of children with ASD, i.e. IQ, language skills, inhibitory control, mental flexibility, and self-control. This is done based on the “Emotion dysregulation in ASD model” by Mazefsky et al. (2013), which highlights ASD related cognitive characteristics that are likely to be of influence on emotion regulation and may contribute to management of emotions (Mazefsky et al. 2013).

## Methods

### Participants

This study included 27 children with ASD (25 boys) and 44 typically developing children (35 boys), matched on age (*M*_ASD_ = 59.48, *SD* = 10.45, *M*_control_ = 55.57, *SD* = 11.17, see Table 1). Socio economic status (SES) did not differ between groups (Table 1). Children with ASD were recruited through the Autism Center Rivierduinen (the Netherlands), the Dutch Autism Association (NVA), and the Dutch Association for Developmental Disorders (Balans). Children from the non-clinical control group were recruited through daycare centers, elementary schools, and postings in public areas in the Netherlands. Parent report versions of the Social Responsiveness Scale (SRS; Constantino and Gruber 2005) and the Childhood Behavior Checklist (CBCL 1,5–5; Achenbach and Rescorla 2000) showed normed sum scores below the clinical cut-off in the non-clinical control group. All parents and/or children were Dutch or English speaking. Children had no neurological conditions, previous head injuries with loss of consciousness, and/or metabolic diseases.

**Table 1:** Demographic characteristics of the ASD group and the non-clinical control group

	ASD (*N* = 27)	Control (*N* = 44)	
	Mean (*SD*)	Mean (*SD*)	Group differences
Age range	43–79 months	41–81 months	*t*(69) = −1.47, *p* = .15
Gender	M = 25, F = 2	M = 35, F = 9	(χ^2^ (1) = 2.18, *p* = .19)
FSIQ	86.89 (22.43)	110.27 (14.61)	*t*(39.69) = 4.83, *p* < .01*
SES^†^ (*N*_ASD_ = 26, *N*_control_ = 42)	2.33 (0.72)	2.61 (0.47)	*t*(38.59) = 1.76, *p* = .09

*Group difference significant at *p* < .05

^†^SES: 1 = low, 2 = medium, 3 = high

### Autism Diagnosis

Diagnosis was provided during a multidisciplinary consensus meeting of child psychiatrists and child psychologists according to the DSM-IV-TR criteria (APA 1994). The diagnostic algorithm of the Autism Diagnostic Interview-Revised (ADI; Le Couteur et al. 2003) was used, which is based on retrospective or current functioning (depending on age) at age 4 to 5 years. Current ASD symptoms were evaluated using the Autism Diagnostic Observation Schedule (ADOS; Lord et al. 2000). Standardized severity scores were calculated according to Gotham et al. (2009) to compare the three different modules of the ADOS that were administered. All children exceeded the diagnostic threshold on both the ADI-R and the ADOS (Table 2).

**Table 2:** ADI and ADOS scores for the ASD group (*N* = *27*)

Scale		Mean (*SD*)
ADI social communication (cut-off = 10)		18.93 (5.87)
ADI communication	Verbal (*N* = 24, cut-off = 8)	14.58 (4.19)
	Non-verbal (*N* = 3, cut-off = 7)	13.20 (0.84)
ADI repetitive behavior (cut-off = 3)		6.41 (3.25)
ADI developmental deviance (cut-off = 1)		4.10 (1.26)
ADOS severity score		8.08 (1.66)

### Child Characteristics

#### Intellectual Functioning

IQ scores of children with ASD were assessed using the test that matched children’s verbal, motor, and developmental level. The majority of children with ASD (24) completed the Dutch Wechsler Nonverbal Scale of Ability (WNV-NL; Wechsler and Naglieri 2006). One child completed the Wechsler Preschool and Primary Scale of Intelligence (WPPSI-III-NL; Wechsler 2006), one child the Snijders-Oomen Nonverbal Intelligence Test (SON-R 2.5–7; Tellegen et al. 1998), and three children the Mullen Scales of Early Learning (MSEL; Mullen 1995). A ratio IQ was computed in case raw scores were outside the standard range for deviation scores by taking the average age equivalents across the subtests, divided by the chronological age in months, multiplied by 100 (Bishop et al. 2011). The non-clinical control group completed the WNV-NL (Wechsler and Naglieri 2006).

#### Inhibition

The Inhibition scale of the Behavior Rating Inventory of Executive Function–Preschool version (BRIEF-P; Gioia et al. 2001) was used to evaluate inhibitory skills (i.e. the ability to suppress impulses and to stop behavior at the right time). The Dutch translation of this parent report questionnaire showed sufficient to high internal consistency, interrater reliability, construct validity, and test–retest reliability indicating suitability for research purposes. Also, it showed adequate convergent discriminant and predictive validity and (Sherman and Brooks 2010; Van der Heijden et al. 2013). The inhibition scale consists of 16 items and higher scores represent lower levels of inhibitory control.

#### Cognitive Flexibility

The Flexibility scale of the Dutch BRIEF-P was used (Van der Heijden et al. 2013). This scale consists of ten items and measures the ability to switch from a situation, activity, perspective, or aspect of a problem to another, if necessary. High scores represent low levels of cognitive flexibility.

#### Self-control

The preschool version of the *Social Skills Rating System* (SSRS; Gresham and Elliott 1990) was used as a measure of self-control, as reported by the primary teacher or mentor. The Self-control scale measures behaviors that emerge in conflict and non-conflict situations, such as taking turns, adequately responding to provocations, and being able to compromise. The scale consists of ten questions that were rated on a 3-point scale. High scores represent low levels of self-control. The SSRS has high internal consistency estimates and moderately high validity indices for total scores (Gresham et al. 2011).

#### Language

The Dutch version of the widely used Peabody Picture Vocabulary Test-III-NL (PPVT-III-NL; Schlichting 2005) was administered to test receptive language skills and vocabulary. Each item of this non-verbal multiple choice test consists of four pictures from which the child is asked to pick the one that corresponds with the examiner’s stimulus word.

### Emotional Expression Measures

#### Locked Box Task (Goldsmith et al. 1999)

**Fig. 1:**
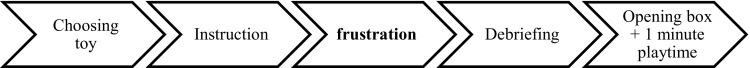
Timeline locked box task (Lab-TAB; Goldsmith et al. 1999)

This task is part of the preschool Laboratory Temperament Assessment Battery (Lab-TAB; Goldsmith et al. 1999). It was designed to evoke frustration by deliberately preventing children from playing with a toy they had chosen for 4 min by handing them a wrong set of keys (see Fig. 1 for a timeline). Recovery was measured by allowing children to play with the desired toy for 1 min directly after unlocking the box. The episode was recorded with video camera’s (JVC Everio GZ-E15) from two angles in order to fully capture the child’s entire body, the box, and the keys. Physiological data from the locked box task were analyzed in 30 s epochs. Of the total 240 s (4 min), the first 30 s were discarded due to the fact that it takes 30 s for the frustration to start (children first need to try the keys to find out that none fit the lock). The majority of children were able to complete the full 4 min (79% of the control children and 67% of ASD children), however some children were not able to finish the 4 min and for example stoop up and tried to walk away from the task, resulting in motion artifacts. In order to prevent bias due to the duration of the task, it was decided to use the epochs that included data from all children, resulting in seconds 30 through 120. The last 120 s of the task could be discarded after analyzing these epochs for children who did complete the full 240 s, revealing comparable data. The mean duration of the task completed was 230 for the control group and 160 s for the ASD group. In total, 90 s of data were included in the analyses. ECG data were manually checked in consultation with the lab-technicians at Leiden University who was blind to group membership of the children. Data were excluded due to motion artifacts that could not be corrected using digital filters.

#### Observational Coding

Children’s emotional coping strategies were coded and categorized as described in Jahromi et al. (2012). In total 14 emotion coping strategies were coded in 10-second intervals as either present or absent (scored as 1 or 0). These 14 coping strategies were assessed independently which means that more than one strategy can occur in the same 10-s interval. Strategies were grouped into three categories and their average inter-rater reliability (expressed in Cohen’s kappa) were (a) constructive strategies (consisting of strategy behaviors such as goal-directed behaviors, orienting to experimenter or parent, and social support seeking); *k* = 0.84, (b) venting strategies (i.e. vocal venting, self-soothing, and self-speech); *k* = 0.82, and (c) avoidance strategies (avoidance, distraction, and alternate strategies); *k* = 0.81. Two independent coders scored all videos. Interrater reliability was monitored continuously in regular consensus meetings. Cohen’s kappa reflect reliability between the two independent coders on 20% of the videos, in percentages the coders reached 93, 96, and 97% agreement on the categories, respectively. Three strategies (disruptive behavior, physical venting, and other-directed comfort-seeking) occurred too infrequent to be included in subsequent behavioral composites or analyses.

### Emotional Experience Measures

#### Physiological Arousal

During the locked box task data were recorded continuously with AcqKnowledge (Version 4.3.1. BIOPAC Systems Inc.). Electrodes were attached, at the top center of the chest, (10 cm below the suprasternal notch) the bottom left, and right of the ribs (10 cm above the bottom of the rib cage). Recordings were acquired through an Electrocardiogram amplifier (ECG100C) and a BIOPAC data acquisition system (MP150 Windows) with a sampling rate of 200 Hz. In AcqKnowledge a 0.5 Hz highpass filter and a 50 Hz notch filter were applied to stabilize the ECG signal. Recorded physiological data was further processed by inspecting the detected R peaks and valid interbeat intervals (IBI) in MATLAB Release 2012b (The MathWorks, Inc., Natick, Massachusetts, United States). Motion artifacts were visually identified and excluded from the data. Heart rate data were summarized in 30-s epochs, in concordance with the behavioral data.

### Procedures

Children attended one research visit accompanied by a parent at the Centre for Autism, Leiden, the Netherlands. Both the ASD group and non-clinical control group underwent the same procedures. Children and parents were prepared with an information brochure and a copy set of the electrodes to familiarize. Before the three electrodes were applied to the chest, the skin was cleaned with alcohol swabs. After attaching the electrodes, children were seated behind a touch screen, which showed a house with objects they could select that moved and played sounds. This was done to familiarize children to the experimental setting and for the electrodes to adapt to the skin. For the locked-box task, children were seated in a high chair behind a table on which four toys were on display. After choosing, the unselected toys were placed out of sight and the chosen toy was directly placed in the box to avoid the child from starting to play. The experimenter explained the task, showed the keys and sat out of direct sight from the child in the back of the room together with the parent who filled out questionnaires and was asked not to respond to the child. If the child asked for help, the experimenter was only allowed to say that the mother was busy. By means of debriefing, the experimenter explained that she had given the wrong set of keys and handed the child the correct key. All elements of the experimental procedure were age appropriate and designed for both verbal and non-verbal children. Finally, teacher report SSRS anonymously numbered questionnaires were given to the parents to hand over to the teachers that best knew their child, which could also be a mentor. A return envelope was included for the teacher to return the questionnaire free of costs.

### Statistical Analyses

ECG data were excluded for one child from the control group and nine children from the ASD group, due to motion artifacts. No further participants were excluded after normality inspections. The excluded children with ASD did not differ from the included children with ASD in IQ (*p* = .63), ADI total score (*p* = .443), or ADOS severity score (*p* = .30). After checking baseline heart rate levels with independent samples *t* tests, a GLM repeated measure analysis was performed with the between-subjects factor group (ASD, control) and the within-subjects factor task (60 s baseline heart rate, 90 s frustration, and 60 s recovery). To further analyze within group differences, paired samples* t* tests were done for the ASD and control group separately. Independent samples *t* tests were used to assess group differences (ASD, control) in self-control (SSRS) and executive functioning (BRIEF-P). For the three coping strategies (Constructive, Venting, and Avoidance) Multivariate Analysis of Variance (MANOVA) was done with Group (ASD, control) as fixed factor. For the ASD group, Pearson correlation coefficients were chosen to assess correlations between cognitive functioning and coping strategies within the ASD group. Finally, two separate backward regressions were performed for the ASD group with the cognitive variables to identify the prominent predictors for coping strategies. Effect sizes are reported as ɳ_*p*_^2^ with 0.01 being a small, 0.06 medium, and 0.14 a large effect (Cohen 1977). Cohen’s *d* effect sizes were calculated for the paired samples *t* tests with 0.2 being a small, 0.5 medium, and 0.8 a large effect.

### Ethics Statement

This study was approved by the Ethical Committee of the Leiden University Medical Center, Leiden, the Netherlands. A written informed consent, according to the declaration of Helsinki, was signed by the parents of all participating children. All tests were completed at the Centre for Autism by a certified child psychologist and trained experimenters who used written protocols detailing all procedures and verbal instructions.

## Results

### Emotional Arousal: Psychophysiological Arousal in Response to Frustration

**Fig. 2:**
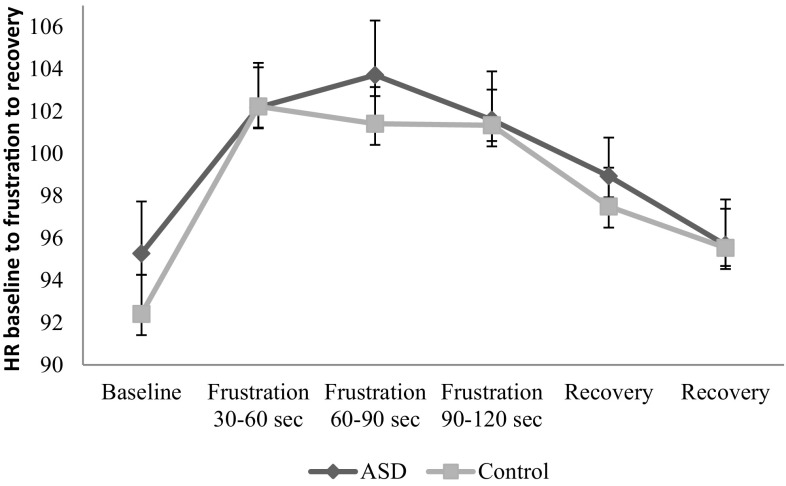
Heart rate (BPM) from baseline to frustration and from frustration to recovery in 30 s epochs. *Error bars* displaying *SE*. At all individual time points there are no significant differences between groups

There was no difference in baseline heart rate between children with ASD (*M* 95.92, *SD* 10.85) as compared to children in the control group (*M* 92.41, *SD* 12.24) (*t*(62) = −1.10, *p* 0.28). Next, group differences in arousal during the locked box task were analyzed. GLM repeated measure analysis with the between-subjects factor group (ASD, control) and the within-subjects factor task (60 s baseline heart rate, 90 s frustration, and 60 s recovery) revealed a significant main effect of Task (*F*(5,305) = 33.49, *p* < .001, ɳ_*p*_^2^ = 0.35), no main effect for Group (*F*(1,61) = 0.16, *p* = .69, and no interaction effect (*F*(5,305) = 0.93, *p* = .46) indicating no difference in the pattern of arousal response across groups. Results are presented in Fig. 2. To further investigate the arousal responsivity for both groups, within group comparisons revealed similar increases and decreases during the locked box task. In other words, arousal increase in response to frustration and arousal reduction during recovery were similar for both groups separately (Table 3).

**Table 3:** Within group increases in emotional arousal (heart rate) during the locked-box task

	ASD	Control
Baseline to frustration 1	*t*(18) = −2.56, *p* = .02*, *d* 0.6	*t*(43) = 7.49, *p* < .01*, *d* 0.6
Baseline to frustration 2	*t*(18) = −3.82, *p* < .01*, *d* 0.7	*t*(43) = −10.95, *p* < .01*, *d* 0.8
Baseline to frustration 3	*t*(18) = −5.00, *p* < .01*, *d* 0.8	*t*(43) = -10.40, *p* < .01*, *d* 0.8
Recovery 1 to recovery 2	*t*(18) = 3.09, *p* < .01*, *d* 0.4	*t*(43) = 2.28, *p* = .03*, *d* 0.2

*Significant at *p* < .05. Effect sizes displayed in Cohens *d*

### Behavioral Regulation: Emotion Coping Strategies

Multivariate Analyses of Variance (MANOVA) with the three strategies (Constructive, Venting, and Avoidance) as dependent variables and Group (ASD, control) as fixed factor, revealed a main effect for Group during the locked box task (*F*(3,67) = 12.19, *p* < .001; Wilk’s Λ = 0.65, ɳ_*p*_^2^ = 0.35). Children with ASD engaged significantly less in Constructive strategies as compared to children in the control group. In contrast, children with ASD showed significantly more Venting than controls and Avoidance as compared to children in the control group. Table 4 shows that the group effect is significant for all three strategies.

**Table 4:** Group comparisons of behavioral emotion coping strategies during the locked-box task

	ASD (*N* = 27) Mean (*SD*)	Control (*N* = 44) Mean (*SD*)	*p*	ɳ_*p*_^2^
Constructive strategies	2.29 (1.06)	2.72 (0.55)	.03*	.07
Venting strategies	1.83 (1.39)	0.98 (1.18)	<.01*	.10
Avoidance strategies	0.84 (0.80)	0.12 (0.22)	<.01*	.32

*Group differences significant at *p* < .05

### Self-control

An independent samples *t* test with the *Self-control* scale from the SSRS as dependent and the two groups (ASD, control) as independent variables revealed that teachers of children with ASD reported significantly less self-control skills (*M* 5.52, *SD* 4.41) than teachers from children in the control group (*M* 15.39, *SD* 3.71) [*t*(64) = 9.80, *p* < .01, Cohen’s *d* 2.8, indicating a large effect].

### Executive Functioning

Independent samples *t* tests with Inhibition and Mental flexibility (as measured with the parent reported BRIEF-P questionnaire) as dependent and the two groups (ASD, control) as independent variables revealed that children with ASD had significantly more problems in inhibitory control (*M* = 35.2, *SD* = 6.2) and mental flexibility (*M* = 20.5, *SD* = 5.4) as compared to children from the control group on inhibitory control (*M* = 24.3, *SD* = 4.5) *t*(67) = −8.48, *p* < .01 and cognitive flexibility (*M* = 13.5, *SD* = 3.4) *t*(67) = −6.68, *p* < .01.

#### Predictors of Coping Strategy

For the ASD group, correlations between the emotion regulation variables (three emotion coping strategies) and child characteristics (IQ, language skills, inhibition, mental flexibility and self-control) were calculated using Pearson correlations. Constructive strategies were positively correlated with IQ (*r*_s_.71, *p* < .01) and Language skills (*r*_s_.48, *p* < .01). Indicating that better general cognitive abilities and more specific, better language skills were associated with the use of more constructive emotion regulation skills. The Avoidance strategies were negatively correlated with IQ (*r*_s_ −.39, *p* < .05), Language skills (*r*_s_ −.41, *p* < .05), and self-control (*r*_s_ −.39, *p* < .05) suggesting that weaker IQ, language, and self-control skills were associated with more avoidance. Finally, venting strategies showed no correlations with any of the cognitive functions.

All variables that were significantly correlated with the emotion coping strategies were entered in linear regression analyses, to identify the most prominent predictors. Two separate backward regressions were performed with total score for Constructive strategies (with the dependent variables IQ and language skills) and total score for Avoidance strategies (with the dependent variables IQ, language skills and self-control) as independent variables. For Constructive strategies a significant model was found, *F*(1,26) = 25.6, *p* < .001, with 50.6% explained variance. This model consisted of one significant predictor, which was IQ (*t* = 5.1, *p* < .001, *β* = .71). In other words, higher IQ predicted being able to deploy more constructive strategies. Also for Avoidance strategies a significant model was found, *F*(1,26) = 5.0, *p* = .03, with 16.7% explained variance. This model consisted of one significant predictor, which was Language skills (*t* = −2.2, *p* = .03, *β* = −0.41). In other words, more compromised language skills predicted more avoidance strategies. In further support of this finding, significant group differences in language skills between children with ASD (*M*_WBQ_ = 84, *SD* = 20) and typically developing children (*M*_WBQ_ = 110, *SD* = 10) were found (*t*(34) = 6.13, *p* < .01).

## Discussion

In this study we investigated emotional arousal and emotion regulation strategies in young children with Autism Spectrum Disorders (ASD). We assessed if children with ASD become under-aroused or over-aroused in response to frustration, and how they cope with these emotions in terms of regulation strategies. Core to the study was the inclusion of physiological, i.e. heart rate measures, in parallel to structured behavior observations, which may provide insight in the responsivity of the arousal system in addition to how emotions are regulated.

Measures of emotional arousal revealed that the pattern of emotional responding in children with ASD was similar to that of typically developing children. There was no difference in the arousal response across groups. For both groups separately, there was an increase in arousal from baseline in response to frustration. In addition, both groups showed that heart rate decreased during recovery of frustration, to similar degrees. In other words, children with ASD did not differ in emotional response (from point to point) in the context of frustration as compared to typically developing children.

In contrast, structured behavioral observations of emotion regulation strategies, which were scored parallel to the arousal measures, showed that children with ASD deployed different strategies, specifically increased use of venting and avoidance behavior compared to typically developing children. Children with ASD showed less constructive (i.e. goal directed) strategies compared to typically developing children. These results are in line with other studies using similar groups and measures, i.e. Jahromi et al. (2012). In this study, children with ASD also showed more self-control problems in the daily life school setting as reported by teachers. This is in line with the observed patterns of emotion coping strategies, as self-control plays an important role in order to be able to regulate emotions in daily life, as also shown in this study. Taken together, our data suggest that while children with ASD show similar intensity of emotional arousal responses, they may have difficulties in behavioral regulation of these emotions. In other words, their coping strategies seem different in contrast to an intact emotional response in the context of frustration. This may point to the role of emotion regulation as a mediating factor in this relationship. Behavior, expression, and experience are all part of emotion regulation (Gross and Thompson 2007), and hence may be differentially affected. Our results suggest dysregulation of behavior in children with ASD (with lowered IQ and language skills) and a lack of self-control in how experienced emotions are expressed to others.

To further investigate the seeming discrepancy between intact emotional responses and the use of different regulation strategies, hypothesized mechanisms that could be of influence on this dysregulation were investigated. These results revealed that being able to deploy constructive strategies, i.e. show goal-directed behaviors, was best explained by one single factor, which was intelligence. In contrast, increased use of less constructive strategies to cope with emotions, i.e. avoidance behavior, was best explained by language ability. Language skills were impaired in children with ASD compared to the typically developing group. Interventions targeting these language impairments should for example include teaching appropriate replacement communicative utterances in particular if a child exhibits excessive tantrums (Koegel et al. 2014). Interestingly, executive functioning (inhibition and cognitive flexibility) was not related to any of the emotion coping strategies. It could be that that null findings regarding executive functioning in (high functioning) individuals with ASD can be explained by a lack in difference between individuals with ASD and typically developing peers (Brady et al. 2017). Results in the current study however do not support this hypothesis, showing more problems in inhibitory control and cognitive flexibility in children with ASD as compared to typically developing children. For future research concerning executive functioning it is important to study this preschool phase of development, because it is hypothesized that executive functioning during this phase has not yet become fully on-line to support regulation strategies (Best and Miller 2010), and that regulation strategies are more dependent on language skills. Thus, language impairments at this point may better explain the use of non-constructive emotion coping behavior in children with ASD. However, because of the prolonged development of executive functioning (Best and Miller 2010), we cannot exclude that these cognitive functions may become increasingly more important for managing experienced emotions over the course of development. Research has shown that executive functions, in particular inhibition, show a first leap in development around age 4 or 5 and continue to improve significantly through the age of 8 years (Best and Miller 2010; Diamond 2013). For future research we recommend taking into account these possible age effects which due to the sample size was not part of this study.

This study has limitations that should be addressed. The recovery in arousal was similar between the control group and ASD group, even though coping strategies were different between the groups. This might be explained however by the manipulation that the experimental locked-box task was unsolvable and children eventually all received the correct key in order to open the locked box, after which they were all allowed to play with the favored toy. Thus, recovery in arousal likely resulted from this manipulation, rather than from type of emotion coping strategy. In future research it would be interesting to investigate the effect of different regulation strategies on the levels of emotional arousal and vice versa, how arousal and coping strategies could influence ASD children’s negative emotion expressions. Since arousal can be considered not only as the cause, but also as the consequence of emotion regulation, these mechanisms are interacting in a dynamic fashion, and are mutually co-dependent on each other. In order to learn more about this dynamic relationship we recommend the use of temporal analyses. The behavioral measures (behavior observations) that were used in the current study are too coarse for this approach and are not suited to follow the quick pace of the arousal in such detail which is necessary for temporal analyses in combination with for example measures such as electromyography. With regard to measures of arousal, the current study included a single heart rate measure as indicator of ANS activity. It is recommended for future research to include a larger repertoire such as heart rate variability and skin conductance level (Mauss and Robinson 2009). Furthermore, even though IQ cannot be separated from the effects of the condition (Dennis et al. 2009), IQ was significantly lower in children with ASD and the current results would be strengthened if this study was replicated in a sample of children without ASD and with matched IQ. Finally, nine children with ASD were excluded due to motion artifacts in the heart rate analyses which could not be filtered from the data. Even though the excluded children did not differ from the other children with ASD on intelligence and symptom severity, the exclusion can be considered a limitation of the study. For future research we recommend the use of wireless heart rate analysis techniques to prevent children to get distracted by the wires that are connected to the electrodes.

In sum, the results of this study show that when it comes to situations that trigger intense/ strong emotions, children with ASD do show emotional arousal responses, but they may rely on less efficient strategies to regulation these emotions (i.e. avoidance), in part driven by lower language abilities. In daily life however, emotional responses are often much less strong. We therefore cannot exclude that an ‘atypical’ arousal response might be found in children with ASD when situations are more subtle and/ or ambiguous. Another factor that may differentially impact the arousal system is the presence of social elements in triggering arousal. A recent study with the same samples of children presented evidence that when it comes to emotional arousal in response to others (so in a social context), that the arousal response of children with ASD is significantly diminished in comparison to typically developing children (Zantinge et al. 2017). Together, this indicates that the emotional arousal response of children with ASD is intact when it comes to their own emotions (although children may have difficulties behaviorally regulating these emotions), however that emotional arousal responses are deviant when it comes to responding to emotions of others. This hypothesis is further supported by a pilot study from Levine and colleagues (2012) who reported equal ANS activity and no increase in salivary cortisol between children with high functioning autism and a comparison group in response to a social stressor (Trier Social Stress Test). These results highlight the importance of integrating objective and sensitive measures of emotional arousal in addition to the behavior that is observed on the outside or reported/observed by parents and professionals. Such deficiencies may significantly impact social functioning. Being able to adequately deal with emotions is important for adaptive social responding (Gross 1999). The preschool phase presents a unique window of development and related opportunities, as strategies to cope with emotions are generally still pre-cognitive at this stage and negative scripts are not yet automated (Greenspan and Shanker 2004). This allows and advocates for early intervention targeted at the earliest stages of behavioral control during emotionally arousing situations, in which stimulation of language development may play an important role in the prevention of avoidance strategies as suggested by our findings (Koegel et al. 2014; Zwaigenbaum et al. 2015).
